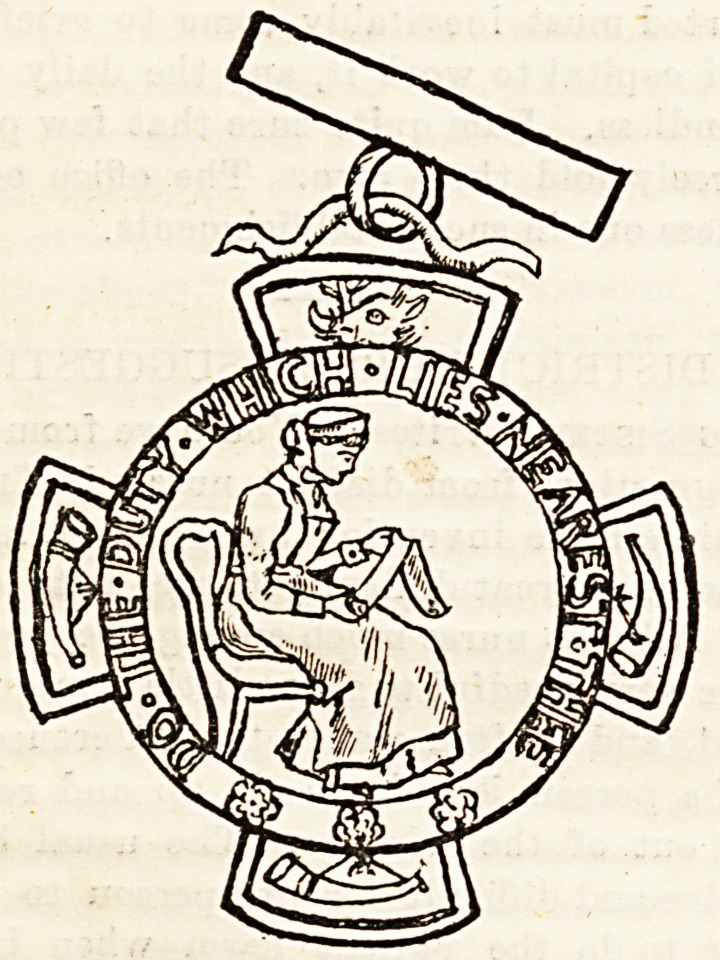# The Hospital Nursing Supplement

**Published:** 1895-08-10

**Authors:** 


"The Hospital" Nursing Mirror.

Being the Extra Nursing Supplement of "The Hospital" Newspaper.

[Contributions for this Supplement should be addressed to the Editor, The Hospital, 428, Strand, London, W.C., and should have the word "Nursing" plainly written in left-hand top corner of the envelope.]

### News from the Nursing World.

### OUR PRINCESS.

The patronage of the Princess of Wales has been given to a scheme to erect a permanent memorial to the late Hans Wilhelm Meyer, of Copenhagen. Her Royal Highness is said to experience particular pleasure at the honour done to her fellow-countryman, and an influential committee has been formed to carry out the scheme. As subscriptions will be accepted from parents whose children have benefited by the discoveries and treatment of this famous doctor (as well as from the medical profession), our nurse readers in all countries will probably hear a great deal about the statue which it is proposed to put up to the memory of the famous Dane.

### HATS ON AND OFF.

Just three years ago a paragraph in The Hospital described the gratification of a small patient because the Prince of Wales took off his hat when he entered a hospital ward. The youngster drew a comparison between His Royal Highness's manners and those of "a committee gentleman" who "fancied 'isself," greatly to the detriment of the latter. Another equally marked example of the Prince's courteous consideration for other people was observed at Marlborough House, when he thoughtfully bid a guest at the garden party resume his hat instead of standing bareheaded in the sun during the distribution of certificates by the Princess.

### THE NURSES' THANKS.

Innumerable verbal thanks and grateful letters have expressed to the secretary of the Royal National Pension Fund the gratitude of nurses for the arrangements by which their enjoyment and comfort were ensured on July 26th. Many are most anxious that the Princess of Wales should be told, through our columns, how much they admire the armlet and certificate, and above all how deeply they appreciate Her Royal Highness's interest in them and their work. The Princess has already expressed her gratification at finding that the afternoon spent at Marlborough House gave so much pleasure to her nurses. "Her kind words expressed to us through the Prince's speech will always live in our remembrance," remarked one, "and we are indeed proud and glad to be numbered amongst the Princess's nurses. We do hope she knows this!" It is pleasant to learn that the rooms recommended to those who stayed in town for the night have in all cases proved satisfactory. Even householders who at first felt sure they could not possibly promise a single room, cheerfully agreed to spare several when they learnt the object for which the nurses were coming to London. In the list of those present at Marlborough House the name of the matron of Lewisham Infirmary was accidentally given instead of that of the assistant matron, Miss Charlotte R. Mill, who had the honour of being specially noticed by the Princess of Wales.

### KILLED BY IGNORANCE.

A striking illustration of the evils of pauper attendants has been given at Chelsea Workhouse, where the recent death of an infant necessitated an inquest. The baby was in the lying-in ward with its mother, and with several other patients was looked after by Nurse Evans during the day and in her absence by an inmate who was engaged as night attendant. In the latter "no experience was required," remarked the nurse at the inquest, as reported by the press. This woman, with neither training nor experience, appears to have been alone with ten patients, and when the infant in question cried she pressed its face against its mother's breast for four or five minutes, after which the child was discovered to be dead from suffocation. The jury agreed that this deplorable result was due to the ignorance of the "night-attendant," the coroner considering it "strange" that someone of experience was not in the ward. His opinion will be echoed by those who are aware that the adjacent infirmary possesses an excellent nurse training school, but to these pupils the lying-in ward at the workhouse is forbidden ground and they must go elsewhere for training in maternity work at the conclusion of three years' general nursing. Yet the workhouse, as well as the probationer, would benefit by the throwing open of the wards. Whilst they appear quite willing to grant them bed linen and mattresses of extravagant quantity and quality, guardians seem often inclined to ignore the claim of the poor for proper attendants at night.

### NURSING OF OUTDOOR PATIENTS.

The Kensington Guardians voted an annual subscription of twenty guineas to the District Nursing Association, after considerable discussion, at the fortnightly meeting of the Board. Out of 611 patients nursed by the association, it was asserted that one-tenth were Poor Law cases. Several of the Guardians spoke very warmly of the valuable work done by the nurses.

### WHO WAS THE NURSE?

The death of a labourer's son, nine years of age, at Plaistow, the other day, was followed by an inquest at which some strange evidence was given. The parents of the boy had not called in medical aid, this being contrary to the creed of the "Peculiar People" to which they belong. The mother, who said she had already lost four other children from bronchitis and consumption, is reported to have remarked that the deceased "had not had good health generally." The only treatment during the illness, which ended fatally, seems to have consisted in the application of oiled rags to the boy's throat. Although the parents' "creed" is put forward to excuse their depriving their offspring of medical treatment, yet it did not prevent their calling in an Elder and a nurse, who "laid their hands on him." We should very much like to know what claim to the title of nurse is possessed by a woman who appears by her action to have acquiesced in the ignorant folly of the parents.

### MACCLESFIELD.

There seems hope for more peaceful times at Macclesfield Infirmary, now that the Press is admitted to the monthly meetings of the governors. Already the local papers comment on the increased order and courtesy observable, and therefore the nursing department may naturally be expected to share the improvements which publicity invariably foster.

### TRAINING AT BRISTOL.

The recent distribution of prizes to the probationers at the Bristol General Hospital took place in the board-room, the chair being taken by Mr. Joseph Storrs Fry. Mr. Proctor Baker (president), Mrs. Baker, Rev. and Mrs. C. Griffiths, Messrs. M. King, Herbert Nash, and C. B. Hare (members of the committee), and Mesdames Naish, Markham Skerritt, May, and Miss Potch (ladies' committee) were also present on the occasion. Nurse Smith (second year nurse) was the winner of the first prize for physiology and anatomy, and Nurse Wales took the second prize. Nurses Cooke and Davis (first year nurses) took first and second prizes respectively in the same subjects. Nurse Wales received the first prize for medical and surgical nursing, and Nurse Bergin the second (both second year nurses). The first and second prizes offered in the same subjects to first year nurses were won by Nurse Davis and Nurse Griffiths. For the next highest aggregate of marks two prizes were given by the matron, Miss Morris, to Nurses M. Simons and Leckie. By an alteration in the rules of the training school it has become compulsory for the nurses to spend three years in hospital before going to private cases.

### SUCCESSFUL ORGANISATION.

The annual report of the Long Sutton Nursing Association is an altogether good one. The accounts show a balance on the right side, and the nurse has been welcomed and appreciated by all who have benefited by her services. The committee are to be congratulated on the successful commencement of their new undertaking.

### OMISSIONS QUICKLY RECTIFIED.

At the beautiful Memorial Hospital recently opened at Crewe the position of the room designed for the operating theatre does not appear to have been approved of by the doctors. It is, therefore, probable that a fresh one will be built, the present one being utilised to accommodate the nursing staff, for whom the allotted quarters are inadequate. The provision of special rooms for night nurses is a necessity not always recognised by building committees, and it is fortunate when omissions and errors in construction can be rectified with the rapidity which has characterised the action of the residents at Crewe.

### NURSES AT SUNDERLAND.

In speaking at the annual court of governors, Mr. James Laing, president of the Sunderland Infirmary, spoke of the excellence of the nursing staff, and Dr. Squance, in an address which attracted much interest, explained the arrangements of that department. Dr. Morgan gave some particulars with regard to the prizes and certificates, which were awarded as follows: Prizes for hygiene to Nurses Moller, Cranswick, McDowell, and Carr; first prize for anatomy, surgical nursing, physiology, and medical nursing to Nurse Moller; second prize to Nurse McDowell. Certificates were obtained by Nurses Dawson, Bone, and Bell.

### PROGRESS AT WIGAN.

The committee of Wigan Infirmary are to be congratulated on their intelligent acknowledgment of the requirements of their nurses. With much good sense and good feeling they are making not only structural additions to the nursing department, but also have sanctioned increased hours off duty for the nurses. A whole day's holiday once a month is now enjoyed by the probationers, whose annual leave has also been extended. The cordial support given by the committee to the matron, Miss Macintyre, in the improvement of the nursing department may well be commended to the attention of those responsible for the management of other infirmaries such as the one at Macclesfield. By the middle of September a new ward for women will be ready for occupation, and at the same time it is hoped to complete the furnishing of eight new bed-rooms and a large sitting-room for nurses.

### "EFFICIENT BUT NOT SUFFICIENT."

The visitors to the Devonport Union have reported that the quantity, not the quality, of the nursing staff is insufficient. There appears to be considerable difficulty in obtaining additional nurses, and the ratepayers would do well to find out the reason of this with a view to remedying it. One of the guardians at a recent meeting is reported as saying "they had done their duty," and moved that the matter should be adjourned for a month. However satisfactory such a motion may appear to those by whom it was carried, it can hardly commend itself to the sick who are still left by the adjournment with an inadequate nursing staff.

### NURSING HOMES.

Requests that The Hospital "will kindly give a notice" to newly-established Homes cannot be complied with unless we have personal knowledge of the institution. The responsibility of even seeming to recommend houses for inspecting which there has been no opportunity, is one we should shrink from for friends in good health, and certainly could not undertake in the case of invalids whose immediate surroundings are as of much importance as the character of their nurses. The selection of both demands experience and knowledge.

### MORE SUBSCRIPTIONS REQUIRED.

The twelfth annual report of the Watford District Nurses shows that 6,547 visits have been paid to 189 patients in the course of the last twelve months by Nurses Bath and Denman. There appears to be constant demand for and appreciation of their services, and it is unfortunate that pecuniary support has not equalled expenditure. According to the local press, funds are urgently needed to maintain the work on its present footing, and the fact is emphasized that out of a population of over 20,000 persons only one hundred subscribe to the District Nursing Fund.

### Elementary Physiology for Nurses.

By C. F. Marshall, M.D., B.Sc., F.R.C.S.

### I.—INTRODUCTION.

If we look at any animal—for instance, a man—we see that there are obvious differences between one part and another, such as the divisions into head, trunk, limbs, &c. These differences are of two kinds—(1) those concerning structure, *i.e.*, what the different parts are made of, which forms the science of anatomy; (2) those concerning function, *i.e.*, what these different parts do and what purpose they fulfil—this constitutes the science of physiology. For instance, the anatomy of the hand deals with the structure of skin, muscles, bones, and nerves, &c.; the physiology of the hand deals with the various things we can do with our hands.

It is impossible to separate physiology from anatomy, for we cannot understand what goes on in an animal unless we know something of its structure. How could we explain the working of a watch to anyone who did not know its structure or anatomy?

We see at once the external divisions into head, neck, trunk, tail and limbs. The trunk is divided into chest or thorax, and abdomen or belly. The shape of the body is formed by the skeleton or framework, which consists of the skull and vertebral column, a bony tube lodging and protecting the brain and spinal cord; the skull also supports the sense organs. The vertebral column or backbone is cut up into rings to allow flexibility, and has bony processes to which the muscles are attached, and which give them firmer attachments, and enable them to act at greater advantage.

The trunk is divided into thorax and abdomen, the division between them being formed by a great transverse muscular partition—the diaphragm. The walls of the thorax are supported by the ribs, while the walls of the abdomen are merely muscular. The thorax contains the heart and lungs; the abdomen contains the stomach, intestines, kidneys, liver, and other organs.

### The Necessity for Food.

Everyone knows that food is necessary to support life. Let us see what happens during starvation. If a man is kept in a pair of scales all day he will steadily get lighter till a meal is taken, when he regains weight. The rate of loss of weight varies according to what the man is doing; during active exercise it is greatest, and less so during rest or sleep. This loss is going on at all times; what it is that is lost, and why this loss occurs, we shall consider later. It is sufficient at present to note that loss of weight is going on always, day and night, waking or sleeping. This waste affects all parts of the body, but at unequal rates. Fat wastes quickest of all; bones, teeth, and brain hardly at all. It is to make up for this loss of weight, affecting all parts at all times, that food is necessary.

### The Digestive System.

Food has to make good the losses of all parts of the body; hence it is clear that great changes must be effected in it before it can do so. There are two chief processes—(1) digestion, by which food is rendered soluble, and so capable of entering the body; (2) assimilation, a further process by which dead food is rendered fit to form part of living tissues.

The digestive organs consist of (1) a long convoluted tube, the alimentary canal, commencing at the mouth and divided into pharynx, œsophagus, stomach, and intestines; (2) the digestive glands, which secrete chemical fluids poured in at various parts to dissolve and effect changes in the food. We can imitate in the laboratory this process of digestion. The non-nutritious part of the food, or waste, is passed away as the fæces.

### The Circulatory System.

We have seen that all parts of the body require food, and we have briefly considered how the food is dissolved in the stomach and intestines. The next problem is to explain how this food is distributed to the various parts of the body. This is done by the circulatory system, which we may compare to a system of waterworks with pipes supplying a number of houses. In the animal body we have a system of pipes laid on to all parts, by which the soluble digested food is taken from the alimentary canal to all parts of the body. These pipes are the blood-vessels.

The heart is a pump by which the fluid is sent round with sufficient rapidity and regularity for the supply of the needs of the various parts of the body. The heart is the most important part of the body, for when it ceases to beat death ensues. We can feel each beat of the heart, each beat of the engine, by feeling one of the pipes at our wrist, the pulse.

The blood is the fluid contained in the pipes or blood-vessels. Every part of the body is wasting, and the various parts are different; therefore each requires different substances for its renewal and repair. This might be accomplished by a separate set of vessels with a separate heart for each part, but this would be a great waste of matter and energy. A far better plan is the actual one, where the blood contains substances required by all parts, and where each part takes from the blood what it wants.

So far we have only traced the blood to the various parts of the body; clearly it cannot stop there or the pipes would burst; moreover, blood from one part is still available for other different parts. Hence there is a second set of vessels which return the blood to the heart; these are the veins. Thus there is a constant stream of blood pumped by the heart into the arteries to all parts of the body, taking nutriment to them, and returning by another set of vessels, the veins, to the heart again. This is the circulatory system.

### "The Hospital" Convalescent Fund.

### THE NURSES' BED.

Application has been made to us on behalf of a nurse requiring change and rest before returning to work after a sharp attack of pneumonia, and we have arranged for her to go to the coast. The case is one to which The Hospital Convalescent Fund is especially applicable, and by its means the nurse will be fitted to rejoin the ranks of useful workers with restored health and energy. Similar appeals as this one are now constantly arising, and therefore more subscriptions are urgently needed for the extension of our work. To help each other is the aim of many nurses, and this most practical way of doing so should commend itself to all. Subscriptions will be gladly received and acknowledged by the hon. secretaries of The Hospital Convalescent Fund, care of Editor, 428, Strand.

### Training for Lecturers.

In October a special course of instruction will commence at Bedford College, Baker Street, for women desiring to qualify themselves for teachers, lecturers, and inspectors. Dr. Louis Parkes will lecture on hygiene, and give practical demonstrations; Dr. Kanthack will teach physiology and bacteriology; Mr. Holland Crompton, chemistry; and Mr. Womack, physics. The course appears to embrace such theoretical and practical teaching as must be specially valuable to trained nurses who desire to qualify themselves for lecturing on health.

### Our Princess's Garden Party.

From a Private Nurse's Point of View.

I was far away at a private case, and felt I ought not to dream of the Princess of Wales's garden party. "Why not take a holiday?" said a friend a fortnight before; "don't go to another case till it's all over." But I resisted the temptation, for to yield meant giving up fees and keeping myself, and perhaps having to wait for a case afterwards if I were to refuse the first one that offered. Luckily, I was sent to some very nice people, and they asked me casually one day what this fund was that the Princess of Wales seemed to be associated with.

When I told them all about it, the young patient's mother remarked, "What a pity you don't belong to it."

"I do," I answered eagerly, and then she soon found out all about my disappointment.

"But why shouldn't you go?" she said pleasantly, "I'll talk to the doctor and see if he will trust me to carry out his orders just for one day."

The patient being really better, the doctor saw no objection to my going to town for the day. "I'll get back as soon as I can," I said gratefully, when in the early dawn I departed, rejoiced at the thoughtful provision which had secured "Return tickets for single fares" for Pension Fund Nurses.

Before ten o'clock I joined the motley gathering inside the Queen's Hall, and, like the others, soon donned my stiffest cap and whitest apron. Oh! what a variety of uniforms there were, the majority being neat and nurse-like.

In due course each of us received the armlet, worked in the Danish colours, and henceforth the badge of the Association. The Princess herself approved, some say she actually designed the device herself.

Then cards of admittance for the afternoon to Marlborough House were methodically given out, whilst the band played, refreshments were enjoyed, and old friends were greeted on all sides. Of course, all of us were tired; some said their backs ached, others had head or leg aches, for there had been much standing about. The fatigue, however, was forgotten when, packed in wagonettes and omnibuses, the procession of some 800 nurses started off to Marlborough House.

I did not know where the "Garden Entrance" might be situated, but the drivers had a larger knowledge, and deposited their loads at the big unpretentious doors. I had passed them oftentimes when I had to take a cab to Victoria Station, without a thought of ever entering them. Still less did I guess that the Royal garden contained a raised walk from which all that passes in that part of the park can be witnessed. In fact, the experiences of the afternoon all combined to show the guests that the Royal party by which they were entertained live in no ignorance of the wants and the ways of "the people."

The great green grass plots, stretching away like fine velvet under foot, were only partially covered by the assembly of nurses.

"Fancy eight hundred seeming to make such little difference," said a cheerful young nurse who stood near me. "But it's just like any quiet country gentleman's house, isn't it? Much nicer than a palace, I am sure." The drilling that had been given in the Queen's Hall in the morning made a very easy matter of the afternoon march past. What fun it was to watch the different styles of nurses, the variety of costumes, and the look of interest on every face.

The Princess seemed to notice all the names as they were given out, one after another, and I am sure she looked at every nurse. It must be rather embarrassing to have hundreds of pairs of eyes staring at you, and it's very good of a Royal lady to smile and look pleasant through the long-drawn-out ordeal. For a whole hour and a quarter did she stand patiently and cheerfully dealing out those certificates, and she never once looked bored.

One of the Duchess of Fife's little children had come out holding the Princess's hand, but getting tired of the show the small personage presently retired, leaving the Princesses Victoria and Maud with their parents. I think it was the Crown Prince of Denmark who sat between the Prince and Princess of Wales, and on her right stood Mr. Burdett, the first founder of the Fund. He handed the certificates to the Princess, and she asked him questions all the time. At least, she seemed to be anxious to understand all about the nurses and their uniforms, and to expect him to know everything. When it was over the Prince made such a nice speech, that I really felt that I knew more about the Fund than ever before. He talked about the great sum of money already invested, and how many nurses had benefited by the Fund. It seemed very plain that the Prince thoroughly understood what he spoke about, and also that the Princess is a really practical president, and not head of the Royal Pension Fund in name only.

When the short speeches were ended, the way to the tea tables was shown by the Princess herself, who walked there with one of the nurses.

The refreshments were most liberal, and fruit and flowers were all of the best. Ices and "cup," and creams, and lots of other good things covered the tables, besides the silver urns of tea; and the Royal servants, taking pattern by their master, were very kind in looking after us. And when the feeding was finished there was still a little time to stroll about or sit under the trees and listen to the band before Big Ben struck five and sounded the signal for departure.

The flowers from the tables were given to those near, and were gladly borne away as mementoes of a day which we shall never forget. I had left my bonnet and cloak at the Queen's Hall, and, finding that other nurses were going back there, a party of us set off together. It was very amusing to see the nurses making their way amongst the ordinary passers-by, very few of whom did more than just look round; but a dear old woman stopped me at a crossing with "Excuse me, nurse, but wherever have you all come from?"

So of course I told her; and when she heard she just dropped a curtsey and said, "Well, I like her, that I do, for having you nurses to tea with her. Bless her pretty face! It makes a body feel envious. But my trade pays better than yours." So I gave the old woman a leaf off my rose, and told her where it had come from, and then left her, quite content with her crossing.

In resuming cloaks and bonnets, everyday duties seemed to be taken up again. The train which bore me back to my patient contained one of the tiredest women in England, but also one of the most contented. My kind employers decided next day that the Princess had earned their thanks for the cheering effect that my recital of my wonderful experiences in Town had on my private patient.

### Where to Go.

Floating Bazaars on board the hospital ship *Albert* will be held on Wednesday and Thursday, August 14th and 15th, at Great Yarmouth, and on Tuesday and Wednesday, August 20th and 21st, at Lowestoft, in aid of the Mission to Deep Sea Fishers. Contributions in gifts or money should be sent to the Secretary, Bridge House, Blackfriars, marked "For the Bazaar."

### Holidays and Health.

[Readers of The Hospital in need of information about health resorts at home or abroad, or desirous of aid in forming holiday plans, are invited to send queries to Editor, 428, Strand, W.C. (marked "Travel" on outside of envelope), which will be answered under this section.]

### AN ORIENTAL HOLIDAY.

Every year some new region of the world is opened up for the benefit of those who like to strike out something original for their holiday plans. This year the tourist is enticed to a part of Europe which very certainly few tourists in the past have ever reckoned within the range of practical comfort-loving people. It is not so long since that Bosnia and Herzegovina were the scene of savage warfare, varied by intervals of so-called peace when "one half of the population was ground down under an iron sway and the other half recognised no other law than the will of a bandit chief." This being so it is a necessary preliminary to any account of these really beautiful regions,and the routes which lead to them, to assure the intending traveller that under the present Government the safety of life and property is absolute, the roads are excellent, the train and diligence service beyond reproach, and the hotels, many of them under Government direction, are fully up to the standard of modern requirements. Three main routes conduct the traveller to Bosnia. The best and quickest, viâ Vienna, Budapest, Szabatka and Brod, leads to the Bosnian capital, Serajevo, but Herzegovina is best approached from Venice across the Adriatic, along the coast of Dalmatia to the port of Metkovic. Although the country appears both remote and inaccessible on the map, the train service has been so far improved that the Bosnian frontier may now be reached in fifty-two hours from London. Its principal attraction is not merely the grandeur of the scenery, fully as that will repay the labour of the journey, but the fact that here, all untouched by the centuries which have transformed the countries of the West, lies a living picture of fourteenth century Oriental life, Eastern customs, Eastern buildings, Eastern colouring, and this within the compass of a ten-pound note from London. From the lively description of these regions by Henri Morse* some idea may be gained of the amazing variety of the country, stretching from fertile plains to wild mountain gorges where the train, ascending often at a gradient of 45 or even 60 degrees, is fitted with cranks to prevent it slipping backwards. Serajevo, in spite of the more modern spirit of the Government and many recent improvements, retains all its old Oriental character. The muezzin may be heard calling the faithful to prayer at the close of day from the hundred minarets and domes, the dervish whirls in his mystic dance, the graceful veils which shroud the features of the Mohammedan lady lend an air of mystery to the streets, and in the bazaars native workmen plying their various trades and a bewildering variety of picturesque wares of every description lie open to view in the uncovered shops. A few hours by rail from Serajevo lies Jajce (pronounced Jaïtze), the old royal city of Bosnia, in many respects the most interesting point in the country. Here are the famous Pliva falls, formed where the lower lake receives by a succession of cascades and rapids the waters of the upper lake, two miles and a-half long; the shores of the lakes are bounded by low hills clothed with forest among which nestle castellated villas built by the Turkish beys for "villeggiatura." The town is built on terraces, among which the numerous minarets are a conspicuous feature, and dominating the whole are the ruins of the royal castle, erected on the model of the Castello del' Uovo, in Naples. The Grand Hotel of Jajce, being under direct Government supervision, is renowned for comfort and cleanliness. The right thing to do is to leave the railway at this point and travel by diligence seven hours through the gorges of the Verbas, some forty-five miles to Banjaluka, whence a return may be made to Vienna, viâ Semmering, by train. The road through the mountains is engineered with admirable skill, so that through the most precipitous passes an almost level course is maintained, and the defiles are extraordinarily wild and picturesque. It would not be easy to exhaust the beauties of this region, extending as it does over an area equal to three fourths of Ireland, and, keeping closely to the main routes, over four hundred miles may be traversed without danger of decent accommodation failing or any real hardship being encountered. To all who wish for a vision of Eastern life without encountering its manifest discomforts the journey may be cordially recommended.

* Bosnia and Herzegovina. By Henri Moser. Drawings by George Scott. (14, Cockspur Street, London.)

### Everybody's Opinion.

[Correspondence on all subjects is invited, but we cannot in any way be responsible for the opinions expressed by our correspondents. No communications can be entertained if the name and address of the correspondent is not given, or unless one side of the paper only be written on.]

### PRIVATE NURSING HOME.

A Lady writes: As superintendent of an old-established and well connected nursing institution, if M. J. (Institute) will take my advice she will think seriously before she undertakes a similar institute or any other kind of one in or near London. The place is overstocked already, and those recently started must inevitably come to grief. It takes a great deal of capital to work it, and the daily necessary expenses are endless. I am quite sure that few pay, and most of them scarcely hold their own. The office of matron is a most thankless one in such establishments.

### A DISTRICT NURSE'S SUGGESTION.

"Nursing Sister" writes: You have from time to time admitted suggestions from district nurses in The Hospital. May I explain a little invention by which I have got over what used to be a great difficulty to me, with chronic cases especially? All who nurse much among the poor know that bed-rests are very needful to give a little comfort and change to an invalid, and it often makes the difference of a good or bad night if a person is able to sit up and read or knit or simply look out of the window. The usual bed rests are very expensive and difficult for one person to arrange, and they are apt to do the patient harm when badly placed. Having experienced all these difficulties for some years in a poor and lonely village, I have hit on the following plan. Net a piece of large-meshed strong netting about 3—4 feet; along one side run a strong webbing with buckles at each end. In the opposite end ordinarily run a rod also with buckles attached. The webbing is fastened across the mattress when the bed is made, nearer or further from the head according to the slope desired; the rod lies by the bed-head; when the patient wishes to sit up, the nurse raises the rod over the bed-head and makes it tight with the buckles, or in the case of a four-poster, the rod is dispensed with and the netting tied to the posts. I have had to do with all manner of bedsteads, whole and broken, but, with a little care and longer or shorter buckles or strings, the bed-rest can be fitted; even where there is no head-board it can be fastened to nails in the wall. Should any district nurse like to see one she can write for prices to Mary Cordelia, nursing sister, Aston Cantlow, Henley-in-Arden.

### Trained Nurses at Bradford.

The medals of the nursing staff at Bradford Infirmary, of which we give illustrations, are of gold and silver respectively, and are particularly handsome in design and workmanship. Introduced in 1892, they are now competed for annually, the winners wearing them for a year, and being entitled to have their names engraved on them. A bronze medal was established early in the present year, and each nurse is presented with one on the completion of her three years' training, together with her certificate, taking both with her when she leaves the infirmary. The matron, Mrs. Magill, is to be congratulated on the improvements she has succeeded in gradually introducing into the system of training at Bradford.

### A Hospital Ship.

The Hospital Mission Ship *Albert* was rendered conspicuous in the harbour at Kiel by its snow-white sail. The presence of this vessel amongst the great warships was designed to familiarise German as well as English sailors with the floating hospital, to which all sick or injured fishermen are welcomed irrespective of nationality. The *Albert*, by permission of the German Emperor, was stationed at the Belle Vue landing-stage at Kiel.

### Appointments.

[It is requested that successful candidates will send a copy of their applications and testimonials, with date of election, to The Editor, The Lodge, Porchester Square, W.]

Newport and Monmouthshire Infirmary.—Miss Hodgkin has been made Matron of this hospital. She was trained at the London Hospital, and was afterwards night superintendent at Cardiff Infirmary, and has taken matron's duty temporarily at the same institution. We wish her every success.

Malta Hospital.—Miss M. J. E. Pinnifer, who was trained at St. Bartholomew's Hospital, and for the last five years has been a nursing sister at the Royal Naval Hospital, Plymouth, has just been appointed to the hospital at Malta. Many good wishes will follow Miss Pinnifer to her new sphere of usefulness.

Manchester Hospital for Consumption and Diseases of the Throat.—Miss Elizabeth Thompson has been appointed Matron of this hospital. She was trained at the Great Western Infirmary, Glasgow, and held the posts of matron of Wincham Cottage Hospital, and of night superintendent at Bradford Infirmary. We wish Miss Thompson every happiness in her new work.

Walsall and District Hospital.—Miss A. Pumphrey has been appointed Matron of this hospital. She was trained at the General Infirmary, Leeds, held the post of sister at Ancoats Hospital, Manchester, and at Queen's Hospital, Birmingham, and that of matron at the Evesham Cottage Hospital. Miss Pumphrey takes many good wishes with her to her new appointment.

West Ham Union Infirmary.—Miss Constance Richard has been appointed Superintendent of Nurses at this infirmary. She received her training at St. Bartholomew's Hospital, was charge nurse and superintendent of nurses at the Metropolitan Asylums Board, North-Eastern Hospital, St. Anne's Road. Miss Richard was afterwards head night nurse at the West London Hospital, and we congratulate her on her present appointment.

### Notes and Queries.

### Queries.

(220) *Training.*—Is a girl of 22 eligible as a probationer? She is strong and healthy. Height, 4 ft 8 in. Weight, 6 st. 11 lbs.—*L. S.*

(221) *Massage.*—Please recommend a book on massage for a nurse?—*A. C. M.*

(222) *Books.*—Where could I get a cheap second-hand "Materia Medica"?—*Nurse.*

(223) *Dispensing.*—Please tell me of books which may be required by a nurse who wants to take up dispensing.—*Nurse Kate.*

(224) *Training.*—Kindly tell me if you think a slight turn in one eye would prevent my being accepted for hospital training.—*A Would-be Nurse.*

(225) *Nursing Abroad.*—What institutions in South Africa pay the passage out of nurses and engage them for three years?—*Africa.*

(226) *Royal Letter.*—Can I obtain another copy of the Princess of Wales' letter of thanks for the screen which the nurses gave Her Royal Highness? I have unluckily lost my Hospital, and I want to replace it.—*A Pension Fund Nurse.*

### Answers.

(220) *Training* (*L. S.*).—We fear the size would be a drawback in hospitals for adults. Write and ask matrons of children's hospitals if they have any rule as to height. You will find a list of training schools in "Burdett's Hospital Annual."

(221) *Massage* (*A. C. M.*).—"The Art of Massage," by Mrs. Creighton Hale, published by Scientific Press.

(222) *Books* (*Nurse*).—Try some of the shops in Booksellers' Row, Strand (near the New Law Courts).

(223) *Dispensing* (*Nurse Kate*).—"British Pharmacopiæ," published by Spottiswoode; Bentley's "Materia Medica," or Mitchell Bruce's; "Luff's Chemistry," "The Art of Dispensing," Ince's Latin Grammar of Pharmacy are all useful books, but you had better consult your teacher as to which he prefers your studying.

(224) *Training* (*A Would-be Nurse*).—There is no universal rule on such a matter. You had better call on the matron, and she and the medical officer who passes the candidates will answer your question.

(225) *Nursing Abroad* (*Africa*).—Do you mean hospitals? When they require nurses they frequently write direct to well-known English training schools for them. You could, however, write yourself and inquire of the matrons of the institutions which are given in "Burdett's Hospital Annual."

(226) *Royal Letter* (*A Pension Fund Nurse*).—The letter appeared as an Extra Supplement in The Hospital of December 12, 1891, and can be obtained by writing to the manager at this office.

## Figures and Tables

**Figure f1:**
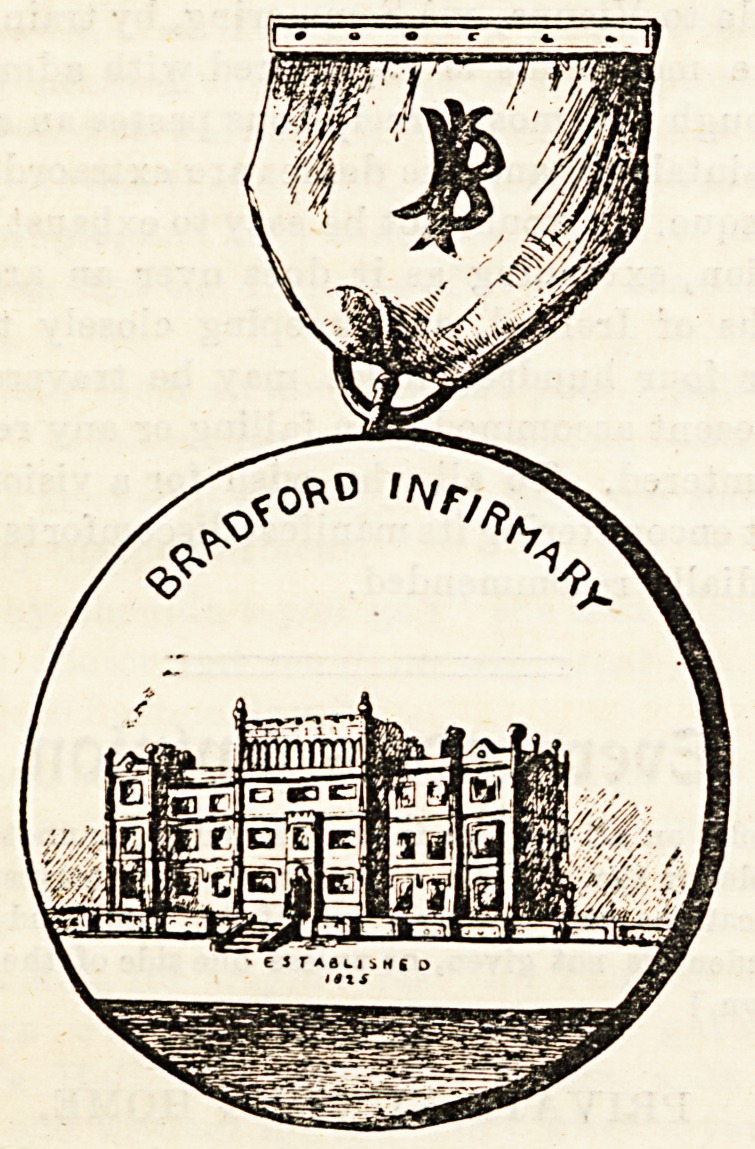


**Figure f2:**